# Genetic polymorphism of merozoite surface proteins 1 and 2 of *Plasmodium falciparum* in the China–Myanmar border region

**DOI:** 10.1186/s12936-019-3003-8

**Published:** 2019-11-19

**Authors:** Cang-Lin Zhang, Hong-Ning Zhou, Quan Liu, Ya-Ming Yang

**Affiliations:** 1 Yunnan Provincial Key Laboratory of Vector-borne Diseases Control and Research, Yunnan Provincial Center of Malaria Research, Yunnan Institute of Parasitic Diseases, Pu’er, 665000 Yunnan China; 2 School of Life Sciences and Engineering, Foshan University, Foshan, 528225 Guangdong China; 3 Key Laboratory of Organ Regeneration & Transplantation of the Ministry of Education, The First Hospital of Jilin University, Changchun, 130021 China

**Keywords:** *Plasmodium falciparum*, Merozoite surface protein, Genetic polymorphism, The China–Myanmar border region

## Abstract

**Background:**

Malaria is a major public health problem in the China–Myanmar border region. The genetic structure of malaria parasite may affect its transmission model and control strategies. The present study was to analyse genetic diversity of *Plasmodium falciparum* by merozoite surface proteins 1 and 2 (MSP1 and MSP2) and to determine the multiplicity of infection in clinical isolates in the China–Myanmar border region.

**Methods:**

Venous blood samples (172) and filter paper blood spots (70) of *P. falciparum* isolates were collected from the patients of the China–Myanmar border region from 2006 to 2011. The genomic DNA was extracted, and the *msp1* and *msp2* genes were genotyped by nested PCR using allele-specific primers for *P. falciparum*.

**Results:**

A total of 215 *P. falciparum* clinical isolates were genotyped at the *msp1* (201) and *msp2* (204), respectively. For the *msp1* gene, MAD20 family was dominant (53.49%), followed by the K1 family (44.65%), and the RO33 family (12.56%). For the *msp2* gene, the most frequent allele was the FC27 family (80.93%), followed by the 3D7 family (75.81%). The total multiplicity of infection (MOI) of *msp1* and *msp2* was 1.76 and 2.21, with a prevalence of 64.19% and 72.09%, respectively. A significant positive correlation between the MOI and parasite density was found in the *msp1* gene of *P. falciparum*. Sequence analysis revealed 38 different alleles of *msp1* (14 K1, 23 MAD20, and 1 RO33) and 52 different alleles of *msp2* (37 3D7 and 15 FC27).

**Conclusion:**

The present study showed the genetic polymorphisms with diverse allele types of *msp1* and *msp2* as well as the high MOI of *P. falciparum* clinical isolates in the China–Myanmar border region.

## Background

Malaria is still one of the most important life-threatening parasitic diseases in tropical and subtropical areas. There were approximate 219 million malaria cases and 435,000 deaths in the world in 2017 [1]. In Southeast Asia, the Greater Mekong Subregion (GMS) is one of the high malarious areas, with the co-existence of different species and emergence of drug-resistant parasites. In Yunnan Province, the most high malarious endemic region in China, annual incidence has decreased from 196/100,000 in 2006 to 0.7/100,000 in 2016, where indigenous malaria transmission is mostly concentrated in Yingjiang County that is adjacent to the Kachin State of Myanmar [2]. In addition, the malaria cases are also clustered on small spatial scales along the China–Myanmar border, which may be related to climatic, environmental, and ecological factors favoring vector survival [3, 4], as well as to the high malaria endemicity in the adjacent Kachin State of Myanmar.

Genetic diversity of the parasites provides useful information on the parasite populations and control efforts against malaria. Polymorphic genetic markers of *P. falciparum* include the merozoite surface protein 1 (MSP1) and MSP2 that have been used to evaluate the genetic diversity of malaria parasites [5–10]. Based on the sequence analysis of *P. falciparum* isolates from different endemic areas, the *msp1* gene is divided into two allelic types of MAD20 and K1, whereas the highly polymorphic block 2 is represented by three allelic types of K1, RO33 and MAD20 [11]. In contrast, the *msp2* gene is grouped into two different allelic types of 3D7 and FC27 [12–14]. These two polymorphic markers have been used to study the *P. falciparum* population in northeastern Myanmar, suggesting a highly diverse parasite population [15]. Due to the dramatic changes of the malaria situation in Yunnan Province, China, in recent years, this study aimed to investigate the genetic diversity of the *P. falciparum* populations along the China–Myanmar border region using two polymorphic markers MSP1 and MSP2.

## Methods

### Collection of clinical parasite samples

**Fig. 1:**
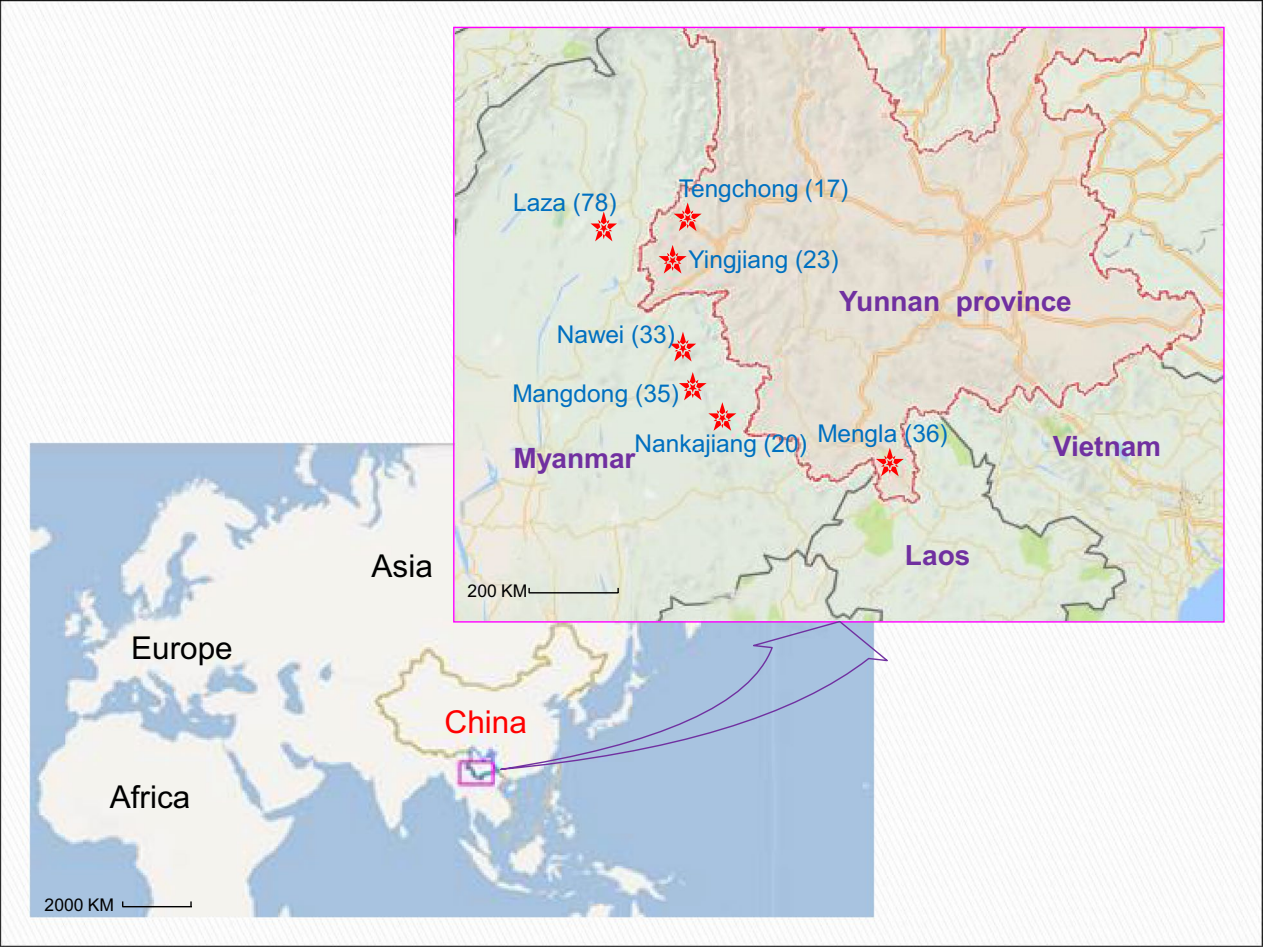
Map of the China–Myanmar border region showing the sampling sites that indicated with red star. The map was prepared by using the website of https://map.baidu.com/

This study was approved by the Ethical Review Board of Yunnan Institute of Parasitic Diseases, China. A total of 242 *P. falciparum* clinical samples were collected from malaria patients attending local hospitals along the China–Myanmar border during 2006–2011. These patients came from Laza, Nawei, Mangdong, and Nankajiang in Myanmar, and Tengchong, Yingjiang and Mengla in Yunnan Province, China (Fig. 1). All patients were diagnosed with *P. falciparum* infection by Giemsa-stained blood smears and microscope examination at the local hospitals, and further confirmed by a nested PCR [16]. Two hundred and fifty microlitres of finger-pricked blood was spotted on the 3 mm Whatman filter paper (GE Healthcare, USA), dried, and stored at 4 °C until further analysis.

### PCR amplification

Genomic DNA of parasite was extracted from the filter paper by using the QIAamp^®^ DNA Mini Kit (Qiagen, Germany). The *msp1* and *msp2* genes were genotyped by a nested PCR using allele-specific primers as described elsewhere [17]. The PCR products were analysed on 2% agarose gel electrophoresis and stained with GoldView (Shanghai, China), whose size was determined using the standard DNA ladder marker (Toyobo, Japan).

The PCR products were sequenced and deposited in the GenBank with accession numbers MG004320–MG004381 for the K1 type, MG004447–MG004517 for the MAD20 type, and MG004518–MG004527 for the RO33 type of *msp1*; MG004219–MG004319 for the 3D7 type and MG004382–MG004446 for the FC27 type of *msp2*.

### Multiplicity of infection

Multiplicity of infection (MOI) was defined as the largest number of alleles at each locus, and single infection was that with only one allele per locus at all of the genotyped loci [15, 18].

### Statistical analysis

All statistical analyses were conducted with the software Statistical Package for Social Sciences (SPSS) version 23.0. The frequencies of the different combinations of alleles in seven studied areas were assessed by Kruskal–Wallis test, and normally distributed continuous data were evaluated by analysis of variance (ANOVA). The Spearman’s rank correlation coefficient test was calculated to assess relationships between MOI and parasite densities or ages in these patients, respectively. The difference was considered statistically significant when *P* value was less than 0.05.

## Results

### General characteristics of included patients

**Fig. 2:**
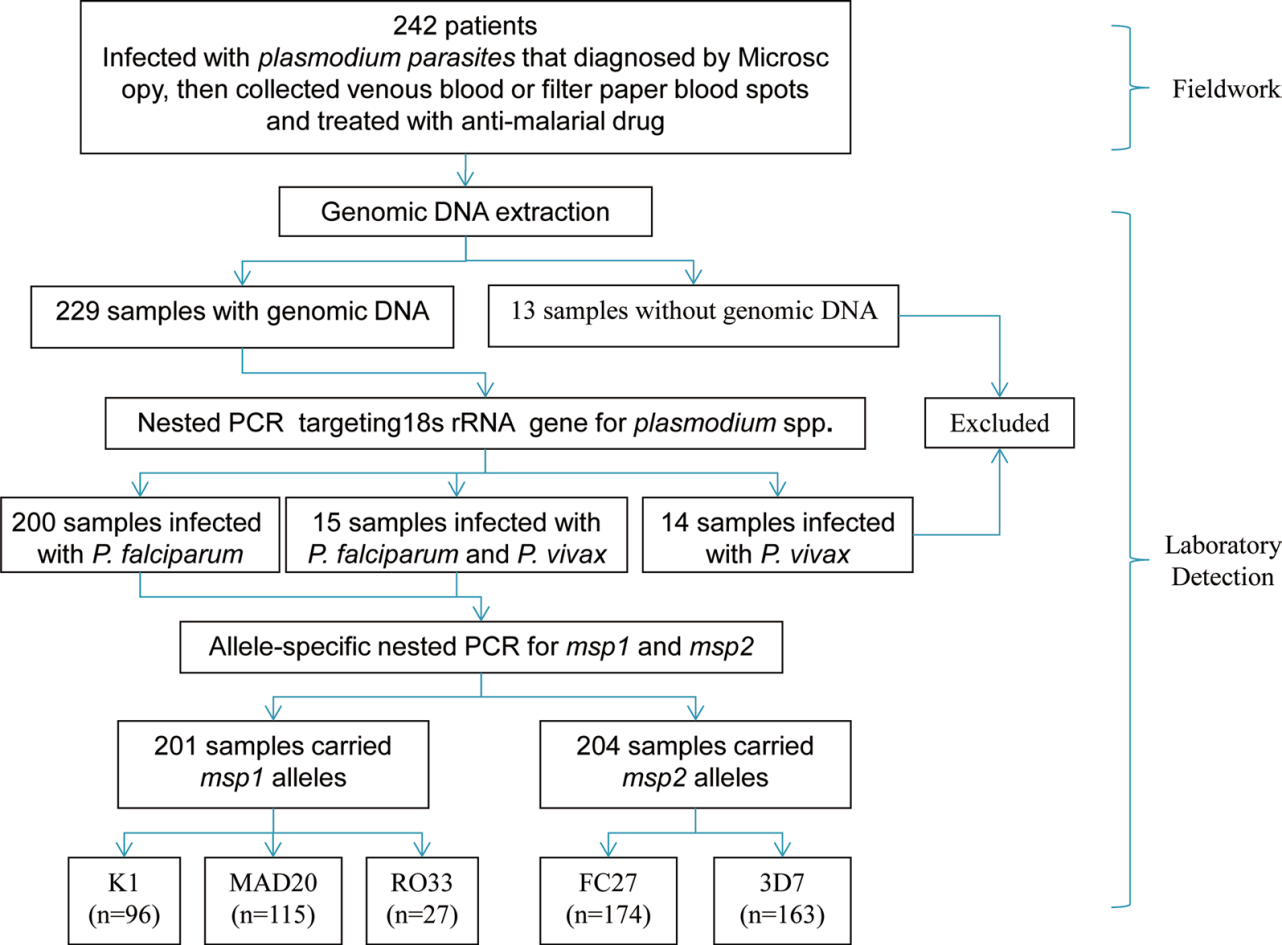
The procedure of samples from fieldwork to laboratory detection

A total of 242 malaria patients were enrolled into this study during 2006–2011. Among these patients, there were 200 patients who were confirmed by a nested PCR to be infected with *P. falciparum*, 14 patients infected with *Plasmodium vivax*, and 15 patients co-infected with *P. falciparum* and *P. vivax*, and 13 patients excluded from the study as the genomic DNA from these patients were not successfully extracted (Fig. 2). All the *P. falciparum* clinical isolates (215 patients) were included in this study. Of these, 65 patients were from Yunnan province, China, and 150 from Myanmar. Sixty-eight patients were children under 19 years, 11 patients were more than 49 years old, and other 136 patients were between 19 and 49 years old.

### Allelic polymorphism of *msp1* and *msp2*

**Fig. 3:**
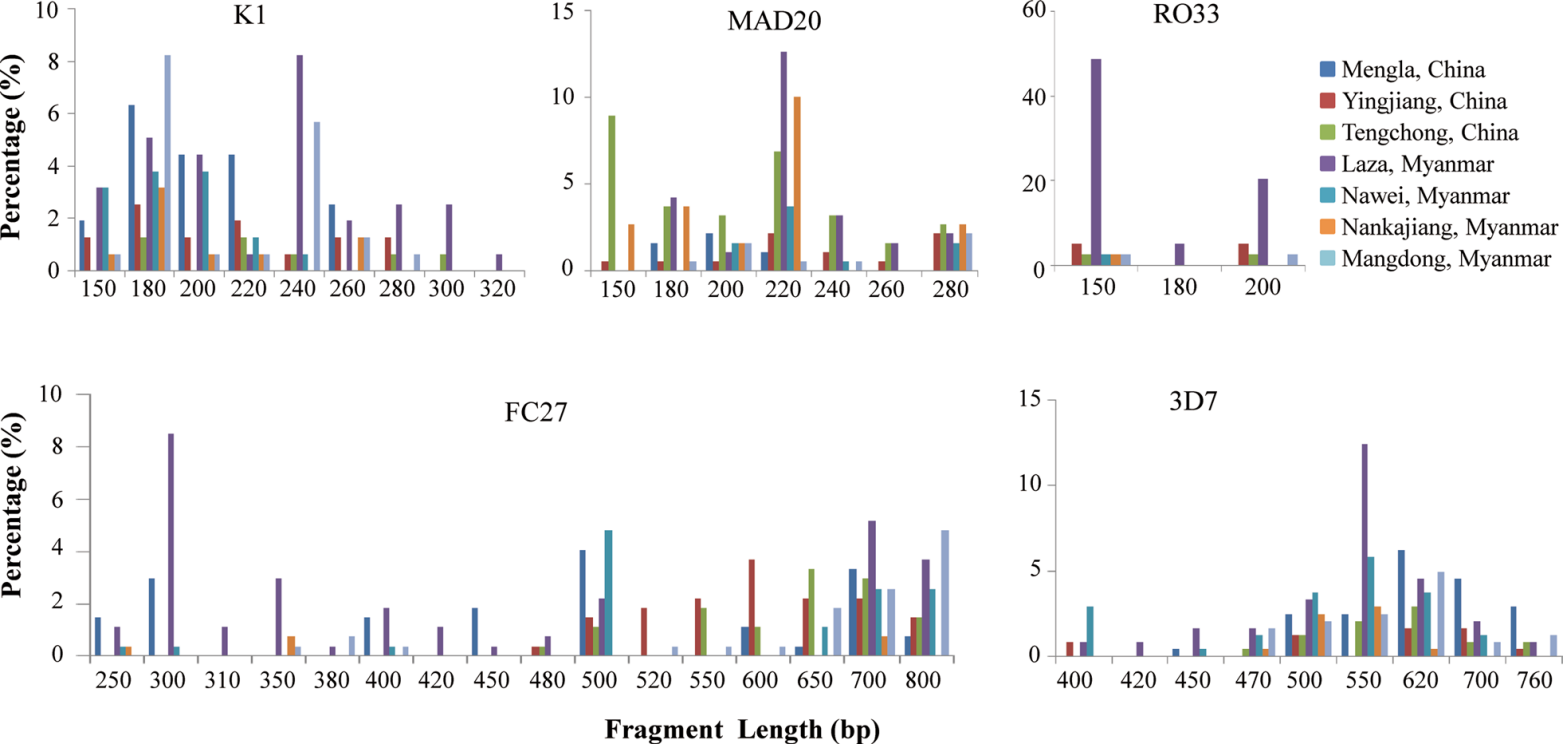
Prevalence of *msp1* and *msp2* alleles classified by fragment length (in base pairs) from allele-specific nested-PCR in different regions of the China–Myanmar border region

Among 215 *P. falciparum* clinical isolates, 201 (93.49%) and 204 (94.88%) samples were genotyped at the *msp1* and *msp2,* respectively. For *msp1* gene, the MAD20 family was dominant (53.49%) and included 7 different band sizes (120–280 bp), followed by the K1 family (44.65%) that included 9 different band sizes (150–320 bp), and the RO33 family (12.56%) that contained 3 different band sizes (150–200 bp) (Table 1, Fig. 3). The alleles with a high frequency for MAD20, K1 and RO33 were 180 bp (30.38%), 180 bp (30%) and 150 bp (64.10%), respectively.

**Table 1:** Prevalence of *msp1* and *msp2* allelic types in the China–Myanmar border region

Allelic types	Mengla, Yunnan	Tengchong, Yunnan	Yingjiang, Yunnan	Laza, Myanmar	Nawei, Myanmar	Nankajiang, Myanmar	Mangdong, Myanmar	Total
	n (%)	n (%)	n (%)	n (%)	n (%)	n (%)	n (%)	n (%)
	n = 28	n = 16	n = 21	n = 77	n = 26	n = 19	n = 28	n = 215
*msp1*								
K1	10 (35.71)	5 (31.25)	5 (23.81)	16 (20.78)	9 (34.62)	7 (36.84)	13 (46.43)	65 (30.23)
MAD20	11 (39.29)	9 (56.25)	10 (47.62)	28 (36.36)	8 (30.77)	7 (36.84)	11 (39.29)	84 (39.07)
RO33	0	1 (6.25)	0	15 (19.48)	1 (3.85)	1 (5.26)	1 (3.57)	19 (8.84)
K1/MAD20	7 (25.00)	0	2 (9.52)	8 (10.39)	6 (23.08)	0	2 (7.14)	25 (11.63)
KI/RO33	0	0	0	2 (2.6)	0	0	0	2 (0.93)
MAD20/RO33	0	0	0	2 (2.6)	0	0	0	2 (0.93)
K1/MAD20/RO33	0	0	3 (14.29)	1 (1.3)	0	0	0	4 (1.86)
Negative	0	1 (6.25)	1 (4.76)	5 (6.49)	2 (7.69)	4 (21.05)	1 (3.57)	14 (6.51)
Multiclonal isolates	22 (78.57)	12 (75.00)	13 (61.90)	51 (66.23)	12 (46.15)	3 (15.80)	25 (89.29)	138 (64.19)
Mean MOI	1.83 ± 0.59	1.94 ± 0.68	2.01 ± 1.28	1.82 ± 0.94	1.44 ± 0.58	1.15 ± 0.40	1.98 ± 0.51	1.76 ± 0.85
*msp2*								
FC27	1 (3.57)	2 (12.5)	11 (52.38)	21 (27.27)	1 (3.85)	2 (10.53)	3 (10.71)	41 (19.07)
3D7	3 (10.71)	0	0	8 (10.39)	6 (23.08)	12 (63.16)	1 (3.57)	30 (13.95)
FC27/3D7	23 (82.14)	13 (81.25)	9 (42.86)	43 (55.84)	19 (73.08)	3 (15.79)	23 (82.14)	133 (61.86)
Negative	1 (3.57)	1 (6.25)	1 (4.76)	5 (6.49)	0	2 (10.53)	1 (3.57)	11 (5.12)
Multiclonal isolates	26 (92.86)	14 (87.50)	19 (90.48)	52 (67.53)	21 (80.77)	3 (15.79)	20 (71.43)	155 (72.09)
Mean MOI	3.27 ± 0.99	3.29 ± 1.20	2.57 ± 1.21	1.86 ± 1.03	2.63 ± 1.51	1.13 ± 0.38	2.21 ± 1.08	2.21 ± 1.29

For *msp2* gene, the FC27 family (80.93%) showed the higher number of alleles, with 8 different band sizes (250–700 bp) (Table 1, Fig. 3), followed by the 3D7 family (75.81%) that included 9 different band sizes (400–760 bp). The most frequent alleles of FC27 and 3D7 were 700 bp (19.56%) and 550 bp (28.22%), respectively.

**Fig. 4:**
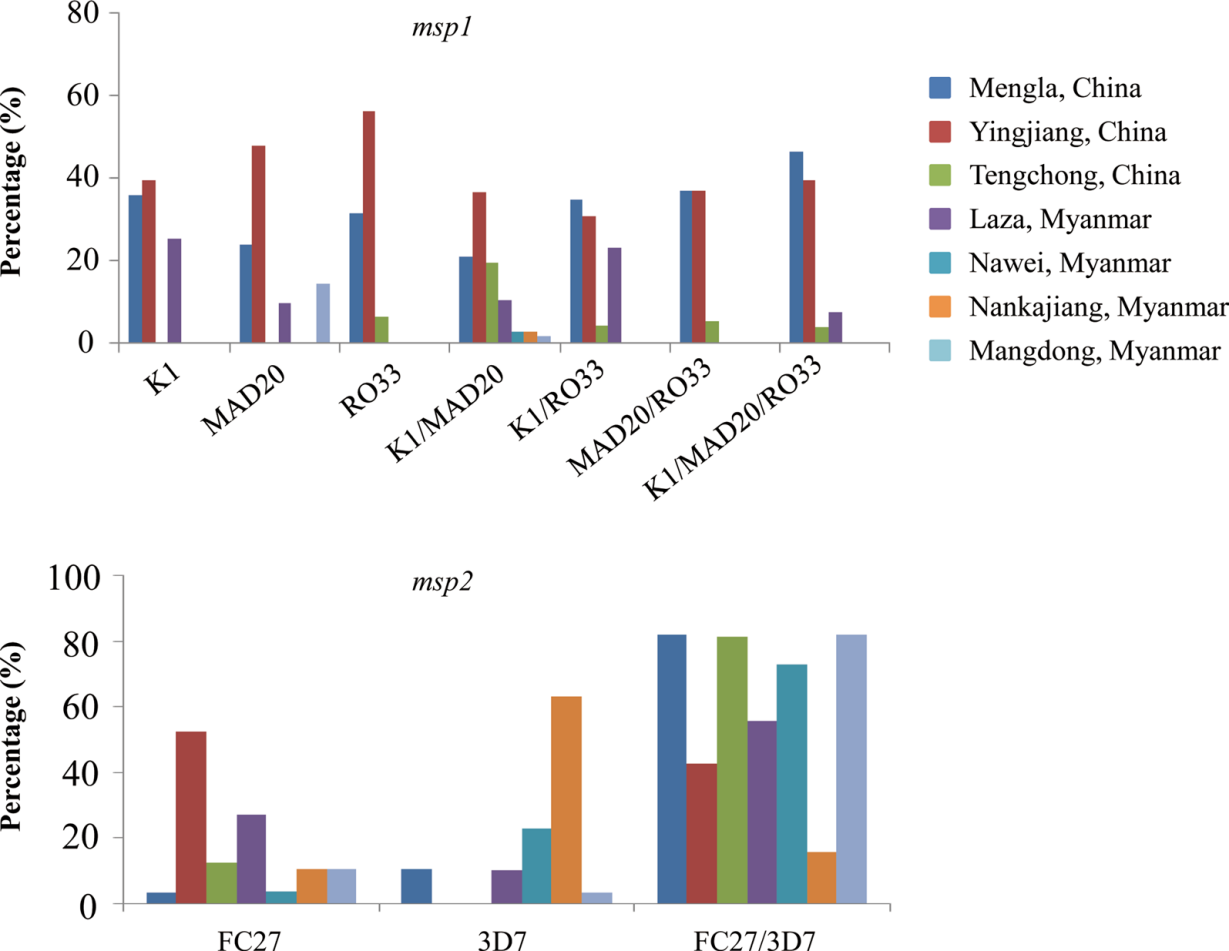
Multiplicity of infection of *msp1* and *msp2* alleles in different regions of the China–Myanmar border region

The total rate of MOI for *msp1* and *msp2* was 64.19% and 72.09%, respectively. The alleles of *P. falciparum clinical isolates* were K1 (30.23%), MAD20 (39.07%), RO33 (8.84%), K1 + MAD20 (11.63%), KI + RO33 (0.93%), MAD20 + RO33 (0.93%), K1 + MAD20 + RO33 (1.86%) for *msp1* and FC27 (19.07%), 3D7 (13.95%), FC27 + 3D7 (61.86%) for *msp2*, respectively, while none of above-mentioned combination alleles of *msp1* (such as K1 + MAD20, KI + RO33, MAD20 + RO33, and K1 + MAD20 + RO33) were be found in the isolates from Tengchong of Yunnan and Nankajiang of Myanmar (Table 1 and Fig. 4). There were a statistical difference in prevalences of the K1, MAD20, and RO33 families and their different MOI of *msp1* (*X*^2^ = 14.478; *P* = 0.025) or FC27, 3D7, and FC27/3D7 alleles of *msp2* (*X*^2^ = 30.617; *P* = 0.000) between the seven studied areas.

### The MOI distribution of allelic families across different parasite density and age groups

Almost all patients were detected to have multiclonal infections, with a mean MOI of 1.76 ± 0.85 for *msp1* and 2.21 ± 1.29 for *msp2*. The MOI values of *msp1* and *msp2* for each parasite density and age groups are summarized in Tables 2 and 3. There was a significant positive correlation between the MOI and parasite density for *msp1* (Spearman’s rank coefficient = 0.208; *P* = 0.002) (Additional file 1: Table S1), but no positive correlation for *msp2* (Spearman’s rank coefficient = − 0.040; *P* = 0.564) was found. Additionally, no significant correlation between age and MOI (Spearman’s rank coefficient = − 0.12; *P *= 0.08 for *msp1* and Spearman’s rank coefficient = 0.007; *P *= 0.917 for *msp2*) was found. Furthermore, there was a significant difference of the MOI for the *msp1* gene among the groups with different parasite densities (F = 2.588; *P *= 0.028), while no significant difference for *msp2* (F = 0.245; *P *= 0.942) or for the MOI of *msp1* (F = 0.443; *P *= 0.818) and *msp2* (F = 0.433; *P *= 0.825) among groups with different ages were found.

**Table 2:** Distribution of *msp1* and *msp2* allelic types of *P. falciparum* among different age groups in the China–Myanmar border region

Allelic types	Age group (years)						Total
	< 9	9–19	19–29	29–39	39–49	≥ 49	n (%)
	n (%)	n (%)	n (%)	n (%)	n (%)	n (%)	
	n = 11	n = 57	n = 70	n = 40	n = 26	n = 11	n = 215
MSP-1							
K1	4 (36.36)	17 (29.82)	23 (32.86)	9 (22.5)	11 (42.31)	1 (9.09)	65 (30.23)
MAD20	2 (18.18)	21 (36.84)	33 (47.14)	13 (32.5)	10 (38.46)	5 (45.45)	84 (39.07)
RO33	0 (0)	7 (12.28)	2 (2.86)	6 (15)	1 (3.85)	3 (27.27)	19 (8.84)
K1/MAD20	4 (36.36)	4 (7.02)	5 (7.14)	8 (20)	3 (11.54)	1 (9.09)	25 (11.63)
KI/RO33	0 (0)	1 (1.75)	0 (0)	1 (2.5)	0 (0)	0 (0)	2 (0.93)
MAD20/RO33	0 (0)	1 (1.75)	0 (0)	1 (2.5)	0 (0)	0 (0)	2 (0.93)
K1/MAD20/RO33	0 (0)	1 (1.75)	2 (2.86)	0 (0)	1 (3.85)	0 (0)	4 (1.86)
Negative	1 (9.09)	5 (8.77)	5 (7.14)	2 (5)	0 (0)	1 (9.09)	14 (6.51)
Multiclonal isolates	4 (36.36)	7 (12.28)	7 (10)	10 (25)	4 (15.38)	1 (9.09)	33 (15.35)
Mean MOI	1.87 ± 0.77	1.88 ± 0.80	1.80 ± 0.90	1.74 ± 0.84	1.44 ± 0.57	1.73 ± 1.18	1.76 ± 0.85
MSP-2							
FC27	1 (9.09)	12 (21.05)	10 (14.29)	13 (32.5)	2 (7.69)	3 (27.27)	41 (19.07)
3D7	2 (18.18)	8 (14.04)	7 (10)	5 (12.5)	8 (30.77)	0 (0)	30 (13.95)
FC27/3D7	8 (72.73)	34 (59.65)	48 (68.57)	20 (50)	16 (61.54)	7 (63.64)	133 (61.86)
Negative	0 (0)	3 (5.26)	5 (7.14)	2 (5)	0 (0)	1 (9.09)	11 (5.12)
Multiclonal isolates	8 (72.73)	34 (59.65)	48 (68.57)	20 (50)	16 (61.54)	7 (63.64)	133 (61.86)
Mean MOI	2.06 ± 1.23	2.09 ± 1.14	2.45 ± 1.29	2.16 ± 1.24	2.04 ± 1.39	2.28 ± 1.72	2.21 ± 1.29

**Table 3:** Distribution of *msp1* and *msp2* allelic types of *P. falciparum* among different parasite densities in the China–Myanmar border region

Allelic type	Parasite density (no. of parasites/µl of blood)						Total
	< 500	500–1000	1000–2500	2500–10,000	10,000–100,000	≥100,000	n (%)
	n (%)	n (%)	n (%)	n (%)	n (%)	n (%)	
	n = 11	n = 22	n = 44	n = 66	n = 61	n = 11	n = 215
*msp1*							
K1	4 (36.36)	9 (40.91)	18 (40.91)	18 (27.27)	14 (22.95)	2 (18.18)	65 (30.23)
MAD20	5 (45.45)	8 (36.36)	17 (38.64)	29 (43.94)	22 (36.07)	3 (27.27)	84 (39.07)
RO33	0 (0)	0 (0)	2 (4.55)	3 (4.55)	8 (13.11)	6 (54.55)	19 (8.84)
K1/MAD20	2 (18.18)	5 (22.73)	2 (4.55)	9 (13.64)	7 (11.48)	0 (0)	25 (11.63)
KI/RO33	0 (0)	0 (0)	0 (0)	0 (0)	2 (3.28)	0 (0)	2 (0.93)
MAD20/RO33	0 (0)	0 (0)	0 (0)	0 (0)	2 (3.28)	0 (0)	2 (0.93)
K1/MAD20/RO33	0 (0)	0 (0)	0 (0)	3 (4.55)	1 (1.64)	0 (0)	4 (1.86)
Negative	0 (0)	0 (0)	5 (11.36)	4 (6.06)	5 (8.2)	0 (0)	14 (6.51)
Multiclonal isolates	2 (18.18)	5 (22.73)	2 (4.55)	12 (18.18)	12 (19.67)	0 (0)	33 (15.35)
Mean MOI	1.32 ± 0.68	1.52 ± 0.73	1.67 ± 0.68	1.91 ± 0.86	1.80 ± 0.95	2.02 ± 0.83	1.76 ± 0.85
*msp2*							
FC27	0 (0)	1 (4.55)	5 (11.36)	17 (25.76)	16 (26.23)	2 (18.18)	41 (19.07)
3D7	1 (9.09)	5 (22.73)	10 (22.73)	6 (9.09)	3 (4.92)	5 (45.45)	30 (13.95)
FC27/3D7	10 (90.91)	15 (68.18)	26 (59.09)	41 (62.12)	38 (62.3)	3 (27.27)	133 (61.86)
Negative	0 (0)	1 (4.55)	3 (6.82)	2 (3.03)	4 (6.56)	1 (9.09)	11 (5.12)
Multiclonal isolates	10 (90.91)	15 (68.18)	26 (59.09)	41 (62.12)	38 (62.3)	3 (27.27)	133 (61.86)
Mean MOI	1.32 ± 0.68	1.52 ± 0.73	1.67 ± 0.68	1.89 ± 0.86	1.80 ± 0.95	2.02 ± 0.83	2.21 ± 1.29

### Sequence analysis of MSP1 and MSP2

A total of 38 different alleles of *msp1* were used for sequence analysis, including 14 for K1 family, 23 for MAD20, and 1 for RO33 (Additional file 2: Figure S1, Additional file 3: Figure S2). All K1-type alleles were found to have a 24-amino acid sequence: SPSSRSNTLPRSNTSSGASPPADA at the 5′-end and 10-amino acid sequence: NEEEITTKGA at the 3′-end. The central variable region always started with SAQ and terminated with SGT containing the difference number of tripeptide repetition, such as SAQ, SGP, and SGT. The diversity of MAD20 family was also caused by differences in repetitions of SGG, SVA and SVT, but the central variable region always started with SKG or SGG and ended with SVA. Only one amino acid sequence type of amino acids LKDGANTQVVAKPAGAVSTQSAKNPPGATVPSGTASTKGAIRSPGAANPSD was identified in the RO33 family. A total of 37 alleles of 3D7 and 15 alleles of FC27 in the *msp2* gene were detected by sequence analysis of the block 3 region (Additional file 4: Figure S3, Additional file 5: Figure S4). The central variable region of 3D7 family included diverse amino acid repeat motifs that contained the different combinations of three amino acids: S, A, and G. The 3D7 family consisted of 12 different amino acids repeats and the 17 single amino acids.

The full sequences of 3D7 family were compared to the reference 3D7 (GenBank accession number X53832) (Additional file 4: Figure S3), showing deletion of 10 or 11 amino acids PKG(K/N)G(E/G/K/Q)VQ(E/K/P)(N/P/S) or PKG(K/N)G(E/G/K/Q)VQ(E/K/P)(N/P/S)N in the reference 3D7, which was common in field isolates from China–Myanmar border region. The dominant genotype of FC27 family was found to have one 32-amino acid ADTIASGSQSSTNSASTSTTNNGESQTTTPTA, followed by a conserved sequence ADTPTAT(E/K), and a two type of tandemly repeated units, SNSPSPPITTTE or SNSRSPPITTTE, repeated from two up to five times. All FC27 family had a 10-amino acid SSSSGNAPNK at the 5′-end and 3-amino acid AP(N/K) or 6-amino acid APNAP(K/N) at the 3′-end (Additional file 5: Figure S4).

## Discussion

The genetic diversity of *P. falciparum* may affect the model of transmission and control strategies for the parasite. The pathogenicity, antigen specificity and anti-malarial drug sensitivity of *P. falciparum* can be associated with their malarial genetic structure [19, 20]. The genetic polymorphism analysis of *P. falciparum* field isolates is necessary and might shed light on the development of control strategies and effective vaccines against *P. falciparum*.

In the present study, a high genetic diversity of *msp1* and *msp2*, including 19 different PCR products for *msp1* (7 MAD20, 9 K1 and 3 RO33) and 17 for *msp2* (8 FC27 and 9 3D7) was found in the *P. falciparum* population in the Myanmar-China border regions. Two PCR products of *msp1* are similar to that in Thailand and western Cambodia [17, 21]. Allele typing for *msp1* showed that MAD20 (115/215, 53.49%) was the predominant allelic type in studied areas, which is consistent with the situations in Thailand, Myanmar, Vietnam, Colombia, Equatorial Guinea, and Yunnan Province, China [15, 22–27]. On the contrary, the K1 family is the most frequent genotype in Laos, Peru, India, Pakistan, Tanzania, Malaysia, and Senegal [28–35]. Allele typing for *msp2* also showed the highest prevalence of FC27 (174/215, 80.93%) and 3D7 (163/215, 75.81%), which is consistent with these situations in Benin [36]. However, 3D7 is the most frequent in Thailand, Myanmar, Colombia, Malaysia, Senegal, India, Equatorial Guinea and Pakistan [16, 21–23, 26, 30, 32, 34, 35, 37, 38]. The overall multiplicity of infection (MOI) of *msp1* and *msp2* was 1.76 and 2.21, respectively. They were similar to those of Thailand and Laos [17, 28], higher than Malaysia, India and Senegal [31, 35, 38], but lower than Ethiopia [39]. The difference may result from the different geographical areas, intensity of malaria transmission and studied populations.

The present study identified 38 alleles of *msp1*, including 14 for the K1 family, 23 for the MAD20 family, and 1 for the RO33 family. This genetic diversity of *msp1* in *P. falciparum* isolates resulted from the different numbers of tripeptide repeat that includes the SAQ, SGP and SGT for K1 and SGG, SVA and SVT for MAD20, which is consistent with the previous studies [15, 22, 28, 30, 40]. Similarly, *msp2* also showed a high genetic diversity, with 37 alleles for the 3D7 family and 15 alleles for the FC27 family. This study showed a highly complicated amino acid repeat motifs in the central variable region of 3D7 alleles that contained the different combinations of S, A and G. There were 4 different continual amino acids repeats, including GASGSA (repeat numbers 2 to 4) [41], GGSGSA (repeat numbers 3 to 9) [41–43], GAVASAGS (repeat numbers 2 to 3) [44] and GAGAVAGS (repeat numbers 3), as well as 2 single amino acid that including GGSA and GAGASAGN, which have also been reported in other studies [14, 41–45]. Several new continual amino acids repeats and single amino acid were also found in this study, including the continual amino acids repeat GAGASGSA (repeat numbers 2), GAGAGAVAGS (repeat numbers 2 to 3), GASGSASGSA (repeat numbers 4 to 5), GAVASAGSRD (repeat numbers 5 to 7), GAGAGAGAVAGS (repeat numbers 2 to 3), PAT (repeat numbers 2 to 6) at the central variable region followed by poly-threonine stretch (repeat numbers 4 to 13) and the intermittent amino acids repeat GAGAGASGSA, GAGASGSAGSGD, GAGAGAGASGSA, as well as the above-mentioned single amino acid except for GGSA at the 3′-end. As reported in previous studies on parasites from Papua New Guinea, Cameroon, Myanmar and China [14, 22, 41, 44], the sequences of the FC27 family of *P. falciparum* isolates in the China–Myanmar border region are conserved at the 3′ and 5′-end, but varied in the number of repeats on SNSPSPPITTTE or SNSRSPPITTTE in the central region.

## Conclusion

The findings of this study have demonstrated that *P. falciparum* clinical isolates in the China–Myanmar border region had a high genetic polymorphism in the *msp1* and *msp2* genes as well as a high multiplicity of infection, suggesting the highly complex population structure of the parasite.

## Supplementary information

**Additional file 1: Table S1.** Multiplicity of infection (MOI) in different groups of age and parasite density.

**Additional file 2: Figure S1.** Alignment of the predicted amino acid sequences of K1 allelic types in *msp1* of all isolates from China–Myanmar border region. The shaded areas indicate the central variable region that compared with the isolate PNG830-048 (GenBank accession number AB502646). Identical residues are indicated by dots. Dashes represent spaces inserted to maximize alignment. Each repeat unit is underlined. The total number of each allele is shown in the last sequences.

**Additional file 3: Figure S2.** Alignment of the predicted amino acid sequences of MAD20 allelic types in *msp1* of all isolates from China–Myanmar border region. The shaded areas indicate the central variable region that compared with the isolate Cam46I-1 (GenBank accession number HM153243). Identical residues are indicated by dots. Dashes represent spaces inserted to maximize alignment. Each repeat unit is underlined. The total number of each allele is shown in the last sequences.

**Additional file 4: Figure S3.** Alignment of the predicted amino acid sequences of 3D7 allelic types in *msp2* of all isolates from China–Myanmar border region. The shaded areas indicate the central variable region that compared with the isolate Tak9 (GenBank accession number X53832). Identical residues are indicated by dots. Dashes represent spaces inserted to maximize alignment. Each repeat unit is underlined. The total number of each allele is shown in the last sequences. The 3D7 family consisted of 12 different amino acids repeats, including GASGSA, GGSGSA, GAVASAGS, GAGAGASGSA, GAGAVAGS, GAGASGSA, GAGAGAVAGS, GASGSASGSA, GAVASAGSRD, GAGASGSAGSGD, GAGAGAGASGSA, and GAGAGAGAVAGS, and the 17 single amino acids comprising of GGSA, GAVAGS, GAGGSGSA, GAGASAGN, GAVASARN, GASGSAGA/S, GASGSAGSGS, GAGAVASAGN, GAGAGASGNA, GAGAGAVASAGN, GAGAGASGSAGSGD, GAGAGASGSAGSRD, GAGAGAGAGAVAGS, GSGAGNGAGNGAGN, GAGAGAGAGAGAVAGS, GAGAGAGASGSAGSGD, and GGSGSAGSGDGNGANP.

**Additional file 5: Figure S4.** Alignment of the predicted amino acid sequences of FC27 allelic types in *msp2* of all isolates in the China–Myanmar border region. The shaded areas indicate the central variable region (GenBank accession number JX885918). Identical residues are indicated by dots. Dashes represent spaces inserted to maximize alignment. Each repeat unit is underlined. The total number of each allele is shown in the last of sequences.

## Data Availability

The data generated during this study are included in this published article and Additional files.

## Abbreviations

MSP1: merozoite surface protein 1
MSP2: merozoite surface protein 2
GMS: Greater Mekong Subregion
MOI: multiplicity of infection

## References

[1] WHO (2018). World malaria report 2018.

[2] Xia ZG, Feng J, Zhou SS (2013). Analysis of malaria epidemic situation in China in 2012. Chin J Parasitol Parasit Dis.

[3] Hui F-M, Xu B, Chen Z-W, Cheng X, Liang L, Huang H-B (2009). Spatio-temporal distribution of malaria in Yunnan Province, China. Am J Trop Med Hyg.

[4] Branch O, Casapia WM, Gamboa DV, Hernandez JN, Alava FF, Roncal N (2005). Clustered local transmission and asymptomatic *Plasmodium falciparum* and *Plasmodium vivax* malaria infections in a recently emerged, hypoendemic Peruvian Amazon community. Malar J.

[5] Mazumdar S, Mukherjee P, Yazdani SS, Jain SK, Mohmmed A, Chauhan VS (2010). *Plasmodium falciparum* merozoite surface protein 1 (MSP-1)-MSP-3 chimeric protein: immunogenicity determined with human-compatible adjuvants and induction of protective immune response. Infect Immun.

[6] Kauth CW, Woehlbier U, Kern M, Mekonnen Z, Lutz R, Mucke N (2006). Interactions between merozoite surface proteins 1, 6, and 7 of the malaria parasite *Plasmodium falciparum*. J Biol Chem.

[7] Mahanty S, Saul A, Miller LH (2003). Progress in the development of recombinant and synthetic blood-stage malaria vaccines. J Exp Biol.

[8] Holder AA, Blackman MJ (1994). What is the function of MSP-1 on the malaria merozoite?. Parasitol Today.

[9] Holder AA, Guevara Patino JA, Uthaipibull C, Syed SE, Ling IT, Scott-Finnigan T (1999). Merozoite surface protein 1, immune evasion, and vaccines against asexual blood stage malaria. Parassitologia.

[10] Genton B, Al-Yaman F, Betuela I, Anders RF, Saul A, Baea K (2003). Safety and immunogenicity of a three-component blood-stage malaria vaccine (MSP1, MSP2, RESA) against *Plasmodium falciparum* in Papua New Guinean children. Vaccine.

[11] Anders RF, McColl DJ, Coppel RL (1993). Molecular variation in *Plasmodium falciparum*: polymorphic antigens of asexual erythrocytic stages. Acta Trop.

[12] Fenton B, Clark JT, Khan CM, Robinson JV, Walliker D, Ridley R (1991). Structural and antigenic polymorphism of the 35- to 48-kilodalton merozoite surface antigen (MSA-2) of the malaria parasite *Plasmodium falciparum*. Mol Cell Biol.

[13] Snewin VA, Herrera M, Sanchez G, Scherf A, Langsley G, Herrera S (1991). Polymorphism of the alleles of the merozoite surface antigens MSA1 and MSA2 in *Plasmodium falciparum* wild isolates from Colombia. Mol Biochem Parasitol.

[14] Smythe JA, Coppel RL, Day KP, Martin RK, Oduola AM, Kemp DJ (1991). Structural diversity in the *Plasmodium falciparum* merozoite surface antigen 2. Proc Natl Acad Sci USA.

[15] Yuan L, Zhao H, Wu L, Li X, Parker D, Xu S (2013). *Plasmodium falciparum* populations from northeastern Myanmar display high levels of genetic diversity at multiple antigenic loci. Acta Trop.

[16] Perandin F, Manca N, Calderaro A, Piccolo G, Galati L, Ricci L (2004). Development of a real-time PCR assay for detection of *Plasmodium falciparum*, *Plasmodium vivax*, and *Plasmodium ovale* for routine clinical diagnosis. J Clin Microbiol.

[17] Snounou G, Zhu X, Siripoon N, Jarra W, Thaithong S, Brown KN (1999). Biased distribution of msp1 and msp2 allelic variants in *Plasmodium falciparum* populations in Thailand. Trans R Soc Trop Med Hyg.

[18] Paganotti GM, Babiker HA, Modiano D, Sirima BS, Verra F, Konate A (2004). Genetic complexity of *Plasmodium falciparum* in two ethnic groups of Burkina Faso with marked differences in susceptibility to malaria. Am J Trop Med Hyg.

[19] Healer J, Murphy V, Hodder AN, Masciantonio R, Gemmill AW, Anders RF (2004). Allelic polymorphisms in apical membrane antigen-1 are responsible for evasion of antibody-mediated inhibition in *Plasmodium falciparum*. Mol Microbiol.

[20] Day KP, Marsh K (1991). Naturally acquired immunity to *Plasmodium falciparum*. Immunol Today.

[21] Gosi P, Lanteri CA, Tyner SD, Se Y, Lon C, Spring M (2013). Evaluation of parasite subpopulations and genetic diversity of the msp1, msp2 and glurp genes during and following artesunate monotherapy treatment of *Plasmodium falciparum* malaria in Western Cambodia. Malar J.

[22] Kang JM, Moon SU, Kim JY, Cho SH, Lin K, Sohn WM (2010). Genetic polymorphism of merozoite surface protein-1 and merozoite surface protein-2 in *Plasmodium falciparum* field isolates from Myanmar. Malar J.

[23] Soe TN, Wu Y, Tun MW, Xu X, Hu Y, Ruan Y (2017). Genetic diversity of *Plasmodium falciparum* populations in southeast and western Myanmar. Parasit Vectors.

[24] Kaneko O, Kimura M, Kawamoto F, Ferreira MU, Tanabe K (1997). *Plasmodium falciparum*: allelic variation in the merozoite surface protein 1 gene in wild isolates from southern Vietnam. Exp Parasitol.

[25] Gomez D, Chaparro J, Rubiano C, Rojas MO, Wasserman M (2002). Genetic diversity of *Plasmodium falciparum* field samples from an isolated Colombian village. Am J Trop Med Hyg.

[26] Chen JT, Li J, Zha GC, Huang G, Huang ZX, Xie DD (2018). Genetic diversity and allele frequencies of *Plasmodium falciparum* msp1 and msp2 in parasite isolates from Bioko Island, Equatorial Guinea. Malar J.

[27] Zhu X, Zhou L, Liu Q, Gao X (1999). Genotype and sequence analysis of merozoite surface protein 1 of *Plasmodium falciparum* isolates in Yunnan province. Chin J Parasitol Parasit Dis.

[28] Khaminsou N, Kritpetcharat O, Daduang J, Charerntanyarak L, Kritpetcharat P (2011). Genetic analysis of the merozoite surface protein-1 block 2 allelic types in *Plasmodium falciparum* clinical isolates from Lao PDR. Malar J.

[29] Chenet SM, Branch OH, Escalante AA, Lucas CM, Bacon DJ (2008). Genetic diversity of vaccine candidate antigens in *Plasmodium falciparum* isolates from the Amazon basin of Peru. Malar J.

[30] Joshi H, Valecha N, Verma A, Kaul A, Mallick PK, Shalini S (2007). Genetic structure of *Plasmodium falciparum* field isolates in eastern and north-eastern India. Malar J.

[31] Bharti PK, Shukla MM, Sharma YD, Singh N (2012). Genetic diversity in the block 2 region of the merozoite surface protein-1 of *Plasmodium falciparum* in central India. Malar J.

[32] Ghanchi NK, Martensson A, Ursing J, Jafri S, Bereczky S, Hussain R (2010). Genetic diversity among *Plasmodium falciparum* field isolates in Pakistan measured with PCR genotyping of the merozoite surface protein 1 and 2. Malar J.

[33] Carlsson AM, Ngasala BE, Dahlstrom S, Membi C, Veiga IM, Rombo L (2011). *Plasmodium falciparum* population dynamics during the early phase of anti-malarial drug treatment in Tanzanian children with acute uncomplicated malaria. Malar J.

[34] Mohd Abd Razak MR, Sastu UR, Norahmad NA, Abdul-Karim A, Muhammad A, Muniandy PK (2016). Genetic diversity of *Plasmodium falciparum* populations in malaria declining areas of Sabah, East Malaysia. PLoS ONE.

[35] Niang M, Loucoubar C, Sow A, Diagne MM, Faye O, Faye O (2016). Genetic diversity of *Plasmodium falciparum* isolates from concurrent malaria and arbovirus co-infections in Kedougou, southeastern Senegal. Malar J.

[36] Ogouyemi-Hounto A, Gazard DK, Ndam N, Topanou E, Garba O, Elegbe P (2013). Genetic polymorphism of merozoite surface protein-1 and merozoite surface protein-2 in *Plasmodium falciparum* isolates from children in South of Benin. Parasite.

[37] Kuesap J, Chaijaroenkul W, Ketprathum K, Tattiyapong P, Na-Bangchang K (2014). Evolution of genetic polymorphisms of *Plasmodium falciparum* merozoite surface protein (PfMSP) in Thailand. Korean J Parasitol.

[38] Atroosh WM, Al-Mekhlafi HM, Mahdy MA, Saif-Ali R, Al-Mekhlafi AM, Surin J (2011). Genetic diversity of *Plasmodium falciparum* isolates from Pahang, Malaysia based on MSP-1 and MSP-2 genes. Parasit Vectors.

[39] Mohammed H, Kassa M, Mekete K, Assefa A, Taye G, Commons RJ (2018). Genetic diversity of the msp-1, msp-2, and glurp genes of *Plasmodium falciparum* isolates in Northwest Ethiopia. Malar J.

[40] Scopel KK, Fontes CJ, Ferreira MU, Braga EM (2005). *Plasmodium falciparum*: IgG subclass antibody response to merozoite surface protein-1 among Amazonian gold miners, in relation to infection status and disease expression. Exp Parasitol.

[41] Basco LK, Tahar R, Escalante A (2004). Molecular epidemiology of malaria in Cameroon. XVIII. Polymorphisms of the *Plasmodium falciparum* merozoite surface antigen-2 gene in isolates from symptomatic patients. Am J Trop Med Hyg.

[42] Thomas AW, Carr DA, Carter JM, Lyon JA (1990). Sequence comparison of allelic forms of the *Plasmodium falciparum* merozoite surface antigen MSA2. Mol Biochem Parasitol.

[43] Felger I, Marshal VM, Reeder JC, Hunt JA, Mgone CS, Beck HP (1997). Sequence diversity and molecular evolution of the merozoite surface antigen 2 of *Plasmodium falciparum*. J Mol Evol.

[44] Jiang GF, Chen PQ, Wang SQ (2002). Genotyping and sequence analysis of the merozoite surface antigen 2 of *Plasmodium falciparum* in Hainan Province, China. Chin J Parasit Dis Con.

[45] Eisen D, Billman-Jacobe H, Marshall VF, Fryauff D, Coppel RL (1998). Temporal variation of the merozoite surface protein-2 gene of *Plasmodium falciparum*. Infect Immun.

