# Supplementary material for: Genetic polymorphism of merozoite surface proteins 1 and 2 of Plasmodium falciparum in the China–Myanmar border region
Source: Malar J. 2019 Nov 19;18:367. doi: 10.1186/s12936-019-3003-8 (PMC6862846; doi:10.1186/s12936-019-3003-8)
Supplement: Supplementary file 1 — Additional file 1: Table S1. Multiplicity of infection (MOI) in different groups of age and parasite density. [file 12936_2019_3003_MOESM1_ESM.docx]

**Table S1.** Multiplicity of infection (MOI) in different groups of age and parasite density.

|  | MOI | |
| --- | --- | --- |
|  | *Msp1* | *Msp2* |
| Age (years) |  |  |
| < 9 | 1.87 | 2.06 |
| 9～19 | 1.88 | 2.09 |
| 19～29 | 1.80 | 2.45 |
| 29～39 | 1.74 | 2.16 |
| 39～49 | 1.44 | 2.04 |
| ≥49 | 1.73 | 2.28 |
| Spearman’s rank coefficient(r) and *p* value | r=-0.12; *p*=0.08 | r=0.007; *p=*0.917 |
|  |  |  |
| parasite density (no. of parasites /µl of blood) | | |
| <500 | 1.32 | 1.32 |
| 500～1,000 | 1.52 | 1.52 |
| 1,000～2,500 | 1.67 | 1.67 |
| 2,500～10,000 | 1.91 | 1.89 |
| 10,000～100,000 | 1.80 | 1.80 |
| ≥100,000 | 2.02 | 2.02 |
| Spearman’s rank coefficient(r) and *p* value | r= 0.208; *p* = 0.002 | r=-0.040; *p* = 0.564 |
